# Clinical evaluation of cochlear implant sound coding taking into account conjectural masking functions, MP3000™

**DOI:** 10.1179/1754762811Y0000000009

**Published:** 2011-11

**Authors:** Andreas Buechner, Andy Beynon, Witold Szyfter, Kazimierz Niemczyk, Ulrich Hoppe, Matthias Hey, Jan Brokx, Julie Eyles, Paul Van de Heyning, Gaetano Paludetti, Andrzej Zarowski, Nicola Quaranta, Thomas Wesarg, Joost Festen, Heidi Olze, Ingeborg Dhooge, Joachim Müller-Deile, Angel Ramos, Stephane Roman, Jean-Pierre Piron, Domenico Cuda, Sandro Burdo, Wilko Grolman, Samantha Roux Vaillard, Alicia Huarte, Bruno Frachet, Constantine Morera, Luis Garcia-Ibáñez, Daniel Abels, Martin Walger, Jochen Müller-Mazotta, Carlo Antonio Leone, Bernard Meyer, Norbert Dillier, Thomas Steffens, André Gentine, Manuela Mazzoli, Gerben Rypkema, Matthijs Killian, Guido Smoorenburg

**Affiliations:** 1 Medizinische Hochschule Hannover (MHH), Carl-Neuberg-Str. 1, 30625 Hannover, Germany; 2 UMC st. Radboud Nijmegen, Postbus 9101, 6500 HB Nijmegen, The Netherlands; 3 Department of Otolaryngology Poznan University of Medical Sciences, ul. Przybyszewskiego 49, Poznan, Poland; 4 Department of Otolaryngology, Medical University of Warsaw, ul. Banacha 1a, Warszawa, Poland; 5 Universitätsklinikum Erlangen, Hals-Nasen-Ohren-Klinik, Waldstraße 1, Erlangen, Germany; 6 St. Salvator Krankenhaus, HNO-Klinik, Gleimstrasse 5, 38820 Halberstadt, Germany; 7 Academisch Ziekenhuis Maastricht, P. Debyelaan 25, 6202 AZ Maastricht, The Netherlands; 8 SOECIC, Institute of Sound and Vibration Research, University of Southampton, SO17 1BJ Southampton, United Kingdom; 9 Universitair Ziekenhuis Antwerpen, Wilrijkstraat 10, 2650 Edegem, Belgium; 10 Istituto di Clinica Otorinolaringoiatrica, Policlinico Universitario “Agostino Gemelli”, L.go A. Gemelli, 8, 00168 Roma, Italy; 11 Medisch Instituut St. Augustinus, Oosterveldlaan 24, 2610 Wilrijk, Belgium; 12 Ospedale Policlinico Consorziale, P.zza G. Cesare, 11, 70124 Bari, Italy; 13 Klinikum der Albert-Ludwigs-Universitaet, Universitaetsklinik fuer Hals-, Nasen- und Ohrenheilkunde und Poliklinik, Killianstrasse 5, 79106 Freiburg, Germany; 14 VUMC Amsterdam, De Boelelaan 1117, 1007 MB Amsterdam, The Netherlands; 15 Charitè Campus Virchow Klinikum, HNO Klinik und Poliklinik, Augustenburger Platz 1, Berlin, Germany; 16 Universitair ziekenhuis Gent, De Pintelaan 185, 9000 Gent, Belgium; 17 Universitätsklinikum Schleswig-Holstein, Klinik fuer Hals-, Nasen, Ohrenheilkunde, Kopf- und Halschirurgie, Arnold-Heller-Strasse 14, 24105 Kiel, Germany; 18 Hospital Insular de Gran Canaria/Materno Infantil, Servicio de Audiologia, Avda.Maritima del Sur, S/N 35016 Las Palmas de Gran Canaria, Spain; 19 CHU La Timone Children Hospital, Rue Saint Pierre 264, 13385 Marseille Cedex 5, France; 20 Hôpital Gui de Chauliac, Service ORL, 80, avenue Augustin, Fliche 34295 Montpellier Cedex 5, France; 21 Ospedale Guglielmo da Saliceto, Via Taverna 49, 29100 Piacenza, Italy; 22 Ospedale di Circolo e Fondazione Macchi, Viale Luigi Borri 57, 21100 Varese, Italy; 23 AMC Amsterdam, Meibergdreef 9, 1105 AZ Amsterdam, The Netherlands; 24 CHU Angers, 4 rue Larrey, 49033 Angers, France; 25 Clinica Universitaria de Navarra, Avda. Pío XII 36, 31008 Pamplona, Spain; 26 Hôpital Avicenne, Service ORL, 125 route de Stalingrad, Bobigny Cédex, France; 27 Servicio de Otorrinolaringologia University Hospital “La Fe” of Valencia, Avda. Campanar, 21, 46009 Valencia, Spain; 28 Otology Institute García-Ibáñez, Dr. Roux 91, 8017 Barcelona, Spain; 29 Uniklinik und Poliklinik fuer HNO-Krankheiten, Petersgraben 4, 4031 Basel, Switzerland; 30 HNO-Universitätsklinik Köln, Audiologie und Pädaudiologie, Kerpenerstr.62, Köln, Germany; 31 Philipps-Universität Marburg, Klinik u. Poliklinik f. HNO-Heilkunde, Deutschhausstraße 3, 35037 Marburg, Germany; 32 U.O.C. di O.R.L, A.O. Monaldi - Napoli, Azienda di Rilievo Nazionale e di Alta Specializzazione, Via L. Bianchi, 80131 Napoli, Italy; 33 Hôpital Saint Antoine, 184 rue du faubourg Saint Antoine, 75012 Paris, France,; 34 Universitätsspital, ORL Klinik, Frauenklinikstrs 24, 8091 Zuerich, Switzerland; 35 Universitätsklinikum Regensburg, HNO-Klinik, Franz-Josef-Strauß-Allee 11, 93053 Regensburg, Germany; 36 Hôpital Hautepierre, Service ORL, Avenue Molière, 67098 Strasbourg Cedex, France; 37 Azienda ospedaliera di Padova, Via Giustiniani 2, 35128 Padova, Italy; 38 Cochlear Europe; 39 Cochlear Europe; 40 “La Palladienne”, Plan Peyrassou, 83890 Besse sur Issole, France

**Keywords:** Cochlear implant, Speech coding, Masking, Spread of excitation, Battery life

## Abstract

Efficacy of the SPEAK and ACE coding strategies was compared with that of a new strategy, MP3000™, by 37 European implant centers including 221 subjects. The SPEAK and ACE strategies are based on selection of 8–10 spectral components with the highest levels, while MP3000 is based on the selection of only 4–6 components, with the highest levels relative to an estimate of the spread of masking. The pulse rate per component was fixed. No significant difference was found for the speech scores and for coding preference between the SPEAK/ACE and MP3000 strategies. Battery life was 24% longer for the MP3000 strategy. With MP3000 the best results were found for a selection of six components. In addition, the best results were found for a masking function with a low-frequency slope of 50 dB/Bark and a high-frequency slope of 37 dB/Bark (50/37) as compared to the other combinations examined of 40/30 and 20/15 dB/Bark. The best results found for the steepest slopes do not seem to agree with current estimates of the spread of masking in electrical stimulation. Future research might reveal if performance with respect to SPEAK/ACE can be enhanced by increasing the number of channels in MP3000 beyond 4–6 and it should shed more light on the optimum steepness of the slopes of the masking functions applied in MP3000.

## Introduction

Present-day cochlear implant electronic circuitry offers high processing capacity while taking little space. Thus, the bandwidth of sound coding is hardly limited but miniaturization of the sound processors requires small power supplies and consequently coding strategies with low energy consumption. Sampling the envelopes rather than the fine structure of the output of a set of contiguous band filters has been proved to be an effective coding strategy for cochlear implants (Continuous Interleaved Sampling, CIS, Wilson *et al*., 1991). The next step in reducing the information to be transmitted, and thus the energy consumption, was implemented in the SPEAK and ACE strategies of the Nucleus implant. Instead of transmitting to the neuronal array the stimulus levels across 22 frequency bands corresponding to 22 electrode locations, the number of stimulus levels transmitted after each sample of the spectral energy distribution was limited to, for example, 10 levels representing the highest levels in that sample. This is referred to as *N*-of-*M* coding, in this example 10 of 22 (Wilson *et al*., 1988, 1995; McKay *et al*., 1991; McDermott *et al*., 1992; Buechner *et al*., 2009). This strategy focused on transferring the most salient sound properties, particularly speech properties, accepting that it suffices to transmit the spectral peaks of the sound.

In the past decade the opposite approach, focusing on hearing properties, has been very successful in sound coding for people with normal hearing. In normal hearing, low-level frequency components of a sound are masked by spectrally adjacent strong components because cochlear excitation introduced by the strong components spreads out to adjacent regions tuned to adjacent frequencies. This implies that one does not need to transfer the information in the components that will be masked, which was implemented in the nowadays widely used MP3 compression strategy (Jayant *et al*., 1993). This approach in signal coding, derived from hearing properties, suggested including spread of excitation and subsequent masking in the ACE coding strategy. However, the new coding was not simply based on rejecting spectral components that are supposed to be masked. In line with the ACE concept, the new coding strategy again recruits *N*-of-*M* spectral components, but rather than taking *N* components with the highest levels it takes *N* components with the highest levels relative to the calculated masked threshold. Thus, components that well exceed the calculated masked threshold will be transmitted. This coding strategy was coined MP3000.

With ACE coding one may expect that two adjacent spectral components are likely to be selected when the spectral energy distribution shows a broad maximum. However, the strongest component will stimulate an array of nerve fibers that might well extend past the adjacent electrode. If subsequently the adjacent electrode is stimulated with the second strongest component, then many nerve fibers at that location might have responded already to the strongest component. With MP3000, it is not likely that an adjacent spectral component will be selected because the level of the second strongest component relative to the calculated masked threshold will be very small. A more distant spectral component will be selected. Thus, MP3000 avoids repetitive stimulation of groups of neurons. It selects components that are dispersed more widely across the spectrum (Nogueira *et al*., 2005, Table 2; Lai and Dillier, 2008).

The present paper reports the results of a European multi-center clinical study in which Nucleus^®^ implant recipients using the SPEAK/ACE strategy were converted to the MP3000 coding strategy. The study included 221 subjects from 37 implant centers. Strategy preference and speech scores were collected. Battery life was recorded.

## Materials and methods

### Selection of spread of excitation parameters and number of components

In the MP3000 approach it was decided not to take a certain masking function or estimate of spread of excitation with electrical stimulation from the literature but to include several preset values as an experimental variable. *A priori*, it was not clear that the electrophysiological and psychophysical measures of masking and spread of excitation available in the literature (Abbas *et al*., 2004; Cohen *et al*., 2004, 2005) would be appropriate. The masking model applied was based on psychophysical estimates for normal hearing in the sense that the masking functions were assumed to follow linear filter slopes in dB per Bark (Zwicker and Feldtkeller, 1967; Zwicker and Fastl, 1990). The frequency scale in Bark is derived from the bandwidth of auditory filters. The band filters in the Nucleus implant closely follow the Bark scale. Also, the current stimulus-level definition of the Nucleus implant closely follows the dB scale, about 5.3 current units per dB. Hence, the slopes were taken as linear in current units per frequency channel. Remarkably, pilot experiments in CI subjects, varying filter slopes, showed that the highest speech scores were found for quite steep slopes (Nogueira *et al*., 2005; Büchner *et al*., 2008). For normal hearing subjects, the low-frequency slope is 27 dB/Bark, quite independent of stimulus level. The high-frequency slope is 27 dB/Bark at low stimulus levels decreasing to 5 dB/Bark at high levels (Zwicker and Feldtkeller, 1967; Zwicker and Fastl, 1990). In the pilot experiments in CI subjects quoted above the highest speech scores were found for even steeper slopes than 27 dB/Bark. In view of those preliminary results, we chose for the present experiment three pairs of low- and high-frequency slopes, independently of stimulus level: 50/37, 40/30, and 20/15 dB/Bark.

Since MP3000 selects spectral components more widely dispersed than SPEAK/ACE (Nogueira *et al*., 2005, Table 2), we expected that less frequency components would be needed to cover the peaks of the spectral energy distribution. Where *N* (of *M*) was 6–14 in the existing SPEAK/ACE fittings of the participating subjects, we reduced *N* to three experimental values of 4, 5, and 6 in the present experiment.

### Study design

Two sessions were planned to find for each subject the optimum number of frequency bands or *channels* from *N* = 4, 5, or 6 and the optimum pair of slopes (from 50/37, 40/30, or 20/15 dB/Bark) in the MP3000 strategy. Starting with nine conditions to choose from was considered to be too demanding for the subjects. Therefore, per subject the optimum number of channels, in terms of subjective preference and speech perception, was first determined for only one pair of slopes, the slopes being assigned at random to the subjects. Second, the optimum pair of slopes was determined for only the optimum number of channels found for each subject in the previous session.

The SPEAK/ACE (A) and MP3000 (B) strategies were compared in a sequential ABABA design. The comparison was conducted for only the optimum combination of number of channels and pair of slopes found in each individual for MP3000. The number of channels in the SPEAK/ACE condition was left unchanged with respect to previous implant usage (8–14). Speech scores were collected as follows: first speech perception was measured for the SPEAK/ACE condition used previously by the subject (A1). After these measurements the subjects received three MP3000 programs with 4, 5, and 6 channels for 4 weeks and the pair of slopes assigned at random. The optimum number of channels was determined from the individual's preference and from speech scores after these 4 weeks. Next, the subjects received three MP3000 programs with the pair of slopes of 50/37, 40/30, or 20/15 dB/Bark and the individual's optimum number of channels for another 4 weeks. Eight weeks after A1 speech scores were collected for the individual's optimum number of channels and the optimum pair of slopes in MP3000 (B1). Subsequently, signal coding was switched back to SPEAK/ACE for 2 weeks after which speech scores A2 were collected. Next, coding was switched back to MP3000 for another 2 weeks and scores B2 were collected. Finally, after 2 more weeks of the SPEAK/ACE and MP3000 strategies at choice, the speech scores A3 were measured for SPEAK/ACE and the individual's preferred strategy was recorded.

In each subject the pulse rate per channel used previously was kept unchanged in SPEAK/ACE during the study. This channel rate was copied into MP3000. Thus, the total pulse rate was markedly lower in MP3000 (4–6 channels) than in SPEAK/ACE (6–14 channels). Battery life was recorded for both strategies.

### Speech tests

The subjects per language were Dutch 32, Dutch-Flemish 19, English 9, French 23, German 58, Italian 26, Polish 26, and Spanish 16. The German and Swiss centers used the same German test. In principle, the different languages do not constitute an experimental problem since the study addresses within-subject comparisons of the coding strategies. In quiet, word tests were applied in all languages. They were scored in either phonemes or words correct (Dutch and Dutch-Flemish: Nederlandse Vereniging voor Audiologie (NVA) monosyllables; English: Consonant Nucleus Consonant (CNC) monosyllables; French: Fournier disyllabic words; German: Freiburg monosyllables; Italian: words; Polish: Pruszewicz monosyllables; Spanish: bisyllabic words). In noise, sentence materials were used at a fixed speech-to-noise ratio of 10 dB for French, Italian, and Polish (French: MBAA2 sentences). For Dutch, Dutch-Flemish, English, German, and Spanish adaptive sentence tests were used. Speech level was varied adaptively in noise fixed at 65 dB Sound Pressure Level (SPL) to find the speech-to-noise ratio at which the score was 50% (Dutch: Plomp sentences, Dutch-Flemish: Leuven Intelligibility Sentence Test (LIST) sentences; English: Bamford Kowal Bench (BKB) sentences; German: Oldenburg sentences; Spanish: Hearing In Noise Test (HINT) sentences).

### T and C levels

Since the MP3000 strategy investigated included fewer channels and, thus, a smaller overall stimulus rate, it was necessary to adjust the T and C levels for the MP3000 strategy in order to obtain a fair comparison to the SPEAK/ACE strategy. T and C levels could be shifted in parallel and, if necessary, tilted. When determining the MP3000 C levels subjects were asked to carefully match the MP3000 loudness to the loudness of the former SPEAK/ACE C levels. Comparing the average T and C profiles across the whole electrode array for the two strategies, parallel upward shifts of 6.9 (s.d. = 8.0) current units for the T levels of MP3000 and 6.8 (s.d. = 6.3) current units for the C levels were found. Some changes in the levels of individual electrodes and in the tilt of the profiles did occur but the result can be well summarized by this parallel shift. One may expect that the shift given above depends on the difference in number of channels between the two strategies. This dependence was found to be very small; only ±1 current unit for both the T and C levels from the smallest difference in number of channels of 8 in SPEAK/ACE and 6 in MP3000 to the largest difference of 14 in SPEAK/ACE and 4 in MP3000.

## Subjects

The study included 221 subjects from 37 European implant centers in 9 countries. Twelve subjects withdrew from the study because they were not willing to take the risk that the change to MP3000 might reduce their hearing performance. Thus, the quantitative data presented in this paper stem from 209 subjects.

The inclusion criteria were:

1. twelve years of age or older. For subjects younger than 18 years, a parental or guardian approval was required;
2. actively using the Freedom™ system for 6 months or longer (CI24RE recipient) or actively using the Freedom BTE system for 1 month or longer (CI24R or CI24M recipient) at entry of the study;
3. ability to read and write in the language of the test materials;
4. sufficient open-set speech perception to allow comparison of strategies;
5. willingness to participate in the study and to comply with all the requirements of the protocol.

The exclusion criteria were:

1. established user of the CIS coding strategy;
2. implanted bilaterally with CI systems;
3. additional handicaps that may prevent participation in the evaluations;
4. unrealistic expectations on the part of the recipient regarding the possible benefits.

Exclusion criteria (1) and (2) were included since the number of controllable experimental variables had to be limited. The present investigation basically addresses the comparison of the new MP3000 strategy to the conventional ACE strategy when selecting fewer channels than available. The CIS strategy is not based on dynamical selection of fewer channels than available. Likewise, monolateral versus bilateral does not touch the major question of the present study. Unrealistic expectations implied, of course, a subjective assessment of the responsible clinician that had to be respected.

The median age was 55 years; the range was 12–85 years. Nine subjects were younger than 18 years. Median severe-to-profound hearing loss (SPHL) duration was 10 years. SPHL duration in 12% of the 209 subjects was larger than 30 years. Twenty-six subjects had pre-lingual SPHL. Still, these subjects had enough open-set speech understanding to complete the speech tests. Ipsi-lateral residual hearing was reported in 10 subjects (5%) and contra-lateral residual hearing in 73 subjects (35%). During the tests contra-lateral hearing was attenuated by inserting an ear plug in the non-implanted ear.

**Figure 1:**
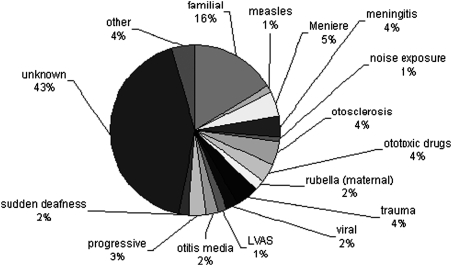
Distribution of etiology of deafness of 209 subjects. LVAS, large vestibular aquaduct syndrome.

**Figure 2:**
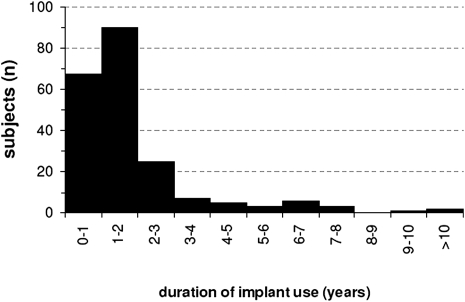
Duration of implant use of 209 subjects before entering the study.

The etiology of deafness corresponded to what one typically finds with 43% unknown origin of deafness and 16% of hereditary/familial origin (Fig. 1). Fig. 2 shows a histogram of the duration of implant use before entering the present study. Median implant use was 1.4 years; the range was 0.2–12.8 years. Implant use in seven subjects was shorter than 6 months, contrary to the intake criterion but accepted.

The study has been conducted according to the guidelines established in the Declaration of Helsinki (Tokyo, 2004). Ethics Committee approvals have been obtained by all participating centers prior to the start of the study. Subjects participating in this study signed a written informed consent prior to any study-related examination or activity.

## Results

### Optimum number of channels

With the preset slope values of 20/15, 40/30, and 50/37 dB/Bark, the optimum number of channels was derived from speech scores and from subjective preference. The results for the speech tests are presented separately for those conducted in quiet and those in noise. The German tests were conducted in noise (58 subjects). The non-German tests presented in quiet were not mandatory. They were completed by 104 of 151 subjects. Table 1 shows the results for both types of tests. Chi-square tests (contingency tables) of the results showed that for both tests the number of channels yielding the highest scores did not depend on the preset slopes (chi-square = 5.9, df = 4, *P* = 0.2 in quiet; chi-square = 2.4, df = 4, *P* = 0.7 in noise). However, for the three pairs of slopes together the sentence-in-noise test showed the highest scores for the highest number of channels (*P* < 0.05 from chi-square, tested against an even distribution).

**Table 1:** Number of subjects with 4, 5, or 6 channels yielding the highest scores in relation to the preset low- and high-frequency slopes of 20/15, 40/30, and 50/37 dB/Bark

Slopes/# channels	4	5	6	Total	Slopes/# channels	4	5	6	Total
20/15	10	8	13	31	20/15	5	7	12	24
40/30	11	19	10	40	40/30	4	3	7	14
50/37	12	8	13	33	50/37	8	3	9	20
Total	33	35	36	104	Total	17	13	28	58

*Left-hand side:* results for the non-German word scores collected in quiet. *Right-hand side:* results for the German sentence test applied in noise.

Asking for preference instead of looking for maximum speech scores also showed that the preset slopes did not affect the preferred number of channels (chi-square = 6.2, df = 4, *P* = 0.2 in quiet; chi-square = 8.2, df = 4, *P* = 0.1 in noise). Yet, taking the results together (Table 2, left-hand panel) there seems to be a small effect: for slopes of 40/30 the preferred number of channels does not seem to follow the general trend of a preference toward higher number of channels (chi-square = 9.8, df = 4, *P* = 0.05). More importantly, however, the trend of a preference toward higher number of channels was quite significant (*P* < 0.05 both in quiet and in noise separately; *P* < 0.005 when combined, from chi-square, tested against an even distribution). Table 2, right-hand panel, shows the relation between preferred number of channels and the number for which the highest speech scores were found. This panel shows that 102 (sum of diagonal elements 25 + 31 + 46) of 162 subjects preferred the number of channels for which the highest speech scores were found. In addition, it shows that 36 subjects preferred a higher and 24 subjects a lower number of channels (the sums of the off-diagonal elements).

**Table 2:** Number of subjects preferring 4, 5, or 6 channels in relation to the preset low- and high-frequency slopes 20/15, 40/30, and 50/37 dB/Bark (left-hand side) and Number of subjects preferring 4, 5, or 6 channels (columns) in relation to the number of channels with the highest speech scores (rows) (right-hand side)

Slopes/# channels	4	5	6	Total	Score/pref	4	5	6	Total
20/15	14	17	39	70	4	25	9	16	50
40/30	23	26	21	70	5	6	31	11	48
50/37	14	20	28	62	6	10	8	46	64
Total	51	63	88	202	Total	41	48	73	162

In summary, across subjects there was little effect of the preset slope values on the number of channels preferred or on those with the highest speech scores. Overall, there was a preference toward the highest number of channels, which was also found for the sentence scores in noise. Whenever the preferred number of channels did not correspond to the number of channels yielding the highest speech score, the clinician asked the subject whether or not the preferred number of channels was preferred above the number of channels yielding the highest speech score. If not the number of channels yielding the highest score was chosen, otherwise the preferred number of channels was kept.

### Optimum pair of slopes

After the second phase of optimizing MP3000 in which the subjects were presented with three pairs of slopes at the optimum number of channels found in the first phase reported above, the results showed that the slopes yielding the highest speech scores did not depend on the optimum number of channels (chi-square = 1.0, df = 4, *P* = 0.9 in quiet; chi-square = 5.9, df = 4, *P* = 0.2 in noise). The same was found for the preferred slopes (chi-square = 5.3, df = 4, *P* = 0.3 in quiet; chi-square = 4.2, df = 4, *P* = 0.4 in noise). However, Table 3 shows that there was a clear preference for the steeper slopes, *P* < 0.001 for both the preferred slopes and those yielding maximum speech scores (from chi-square, tested against an even distribution). This suggests that the slopes preset at the beginning of the experiment, which were about evenly spread, had little effect on the preferred slopes and the slopes yielding maximum speech scores after 8 weeks. This is illustrated in Table 4 showing the preferred slopes in relation to the preset slopes. The number of preferred slopes equal to the preset slopes was 86 (sum of the diagonal elements); 91 preferred slopes were steeper and 32 were shallower than the preset slopes (sum of the off-diagonal elements). In summary, these results showed that the individual's optimum number of channels had no effect on the optimum pair of slopes. Overall, there is a clear preference toward steeper slopes.

**Table 3:** Number of subjects with preferred slopes (left-hand side) and with slopes yielding the highest scores (right-hand side) in relation to the optimum number of channels

# channels/slope	20/15	40/30	50/37	Total	# channels/slope	20/15	40/30	50/37
4	5	22	29	56	4	5	17	19
5	5	21	40	66	5	10	12	27
6	15	34	38	87	6	10	23	32
Total	25	77	107	209	Total	25	52	78

**Table 4:** Preferred slopes in relation to the preset slopes at the beginning of the experiment

Pre/post	20/15	40/30	50/37	Total
20/15	11	26	35	72
40/30	9	33	30	72
50/37	5	18	42	65
Total	25	77	107	209

Whenever the preferred pair of slopes did not correspond to the slopes yielding the highest speech score, the investigator followed the same procedure as for the number of channels described above. The preferred slopes were kept whenever these slopes were preferred above the slopes yielding the highest scores; otherwise the pair of slopes with the highest score was kept.

### Comparison of the speech scores for SPEAK/ACE and MP3000

**Figure 3:**
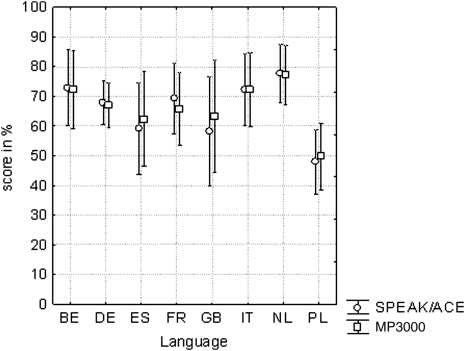
Word scores collected in quiet for the SPEAK/ACE and MP3000 strategies. Number of subjects per language were Dutch (NL) 32, Dutch-Flemish (BE) 19, English (GB) 9, French (FR) 22, German (DE) 57, Italian (IT) 21, Polish (PL) 26, and Spanish (ES) 13. The bars indicate the 95% confidence intervals.

Analysis of variance of the word scores collected in quiet in the ABABA sequence showed no effect of strategy (*P* = 0.5, *F*(1,191) = 0.5). Mean scores were 65.6% for SPEAK/ACE and 66.2% for MP3000. There was a significant effect of order of measurement (*P* < 0.001). However, the effect was small. The scores increased by 3% from the first to the last session. The scores among languages differed significantly (*P* < 0.001) (Fig. 3).

**Figure 4:**
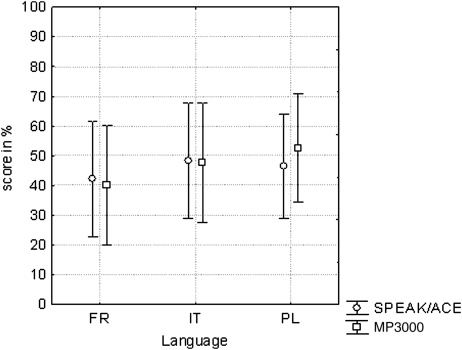
As Fig. 3 but for scores collected in noise at S/N = +10 dB.

**Figure 5:**
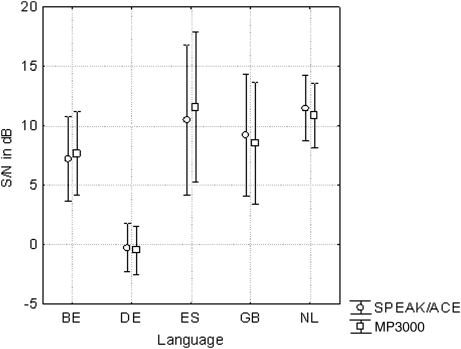
As Fig. 3 but S/N at which sentences in noise reach 50% score (Large confidence intervals for the Spanish language, ES, because the number of subjects was 6 rather than 13).

The word scores collected in noise at S/N = +10 dB (French, Italian, and Polish) showed no effect of strategy (*P* = 0.6, *F*(1,65) = 0.3). Mean scores were 45.7% for SPEAK/ACE (A) and 46.8% for MP3000. There was no change of the scores over time (*P* = 0.5). Also, there was no effect of language (*P* = 0.6): the mean scores for the three languages were 41–50% (Fig. 4). The adaptive sentence tests (Dutch, Dutch-Flemish, English, German, and Spanish) also showed no effect of strategy (*P* = 1.0, *F*(1,118) = 0.0). Mean S/N values were +7.6 dB both for SPEAK/ACE and MP3000. The only significant effect was due to language (*P* < 0.001). Four tests showed thresholds at S/N = +9 to +11 dB; the German threshold was about 0 dB (Fig. 5). It is interesting to note that there was no effect of strategy both at 0 dB and at about 10 dB S/N. The interaction between the factors language and strategy was insignificant (*P* = 0.5, *F*(4,118) = 0.8).

Finally, the question was addressed whether or not the number of channels and pair of slopes affected the scores for the MP3000 strategy. This would imply a between subjects comparison because only one combination of number of channels and pair of slopes was tested in each subject. However, the problem of intersubject differences and different languages (speech tests) in between subject comparisons could be reduced by analyzing the effect of number of channels and pair of slopes on the difference between the MP3000 and SPEAK/ACE scores. The word scores collected in quiet showed no statistically significant effect of number of channels (*P* = 0.7, *F*(2,204) = 0.29) and no significant effect of pair of slopes (*P* = 0.08, *F*(2,204) = 2.5). For slopes there was a trend of somewhat (+3%) higher scores for the 50/37 dB/Bark combination. Also, the word scores collected in noise at S/N = +10 dB showed no statistically significant effect of number of channels (*P* = 0.9, *F*(2,67) = 0.13) and no significant effect of pair of slopes (*P* = 1.0, *F*(2,67) = 0.02). Finally, the sentence thresholds showed no statistically significant effect of number of channels (*P* = 0.7, *F*(2,126) = 0.36) but there was a statistically significant effect of pair of slopes (*P* = 0.01, *F*(2,126) = 4.4). In the 20/15 dB/Bark condition, we found about a 3.5 dB higher (i.e. worse) S/N for the MP3000 strategy than for the SPEAK/ACE strategy. There was no difference in S/N for the steeper slopes. In summary, there was no effect of the number of channels in MP3000 (4, 5, or 6) and there was a tendency of better results for the steeper slopes.

### Preference scores for SPEAK/ACE and MP3000

**Figure 6:**
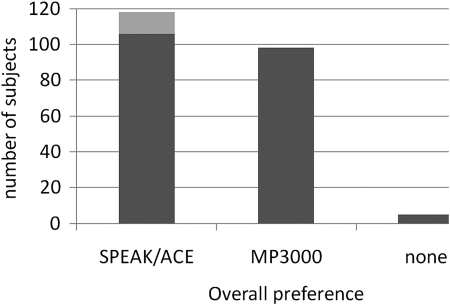
Overall preference for one of the coding strategies. The number of subjects who withdrew from the study (12) has been added to the ACE/SPEAK bar (*n* total = 221).

After the ABABA sequence of speech scores, the study was completed by recording at A3 the overall preferred strategy and preference in quiet and in noise separately. Between B2 and A3 the subjects could use both strategies for 2 weeks. Fig. 6 shows the overall preference. The 12 subjects who withdrew from the study because they did not want to rely on the experimental MP3000 coding in their daily life are added to the SPEAK/ACE bar. There is no statistically significant difference between the number of subjects preferring SPEAK/ACE (106 + 12) and the number preferring MP3000 (98), even when adding the 12 subjects who withdrew from the study (*P* = 0.2, chi-square against even distribution). Only five subjects could not indicate a preference.

**Figure 7:**
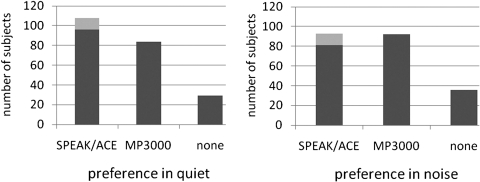
Preference for one of the coding strategies in quiet (left-hand panel) and in noise (right-hand panel). The number of subjects who withdrew from the study (12) has been added to the ACE/SPEAK bar (*n* total = 221).

Asking specifically for preference in quiet and in noisy conditions, there was a larger number of subjects that could not indicate a preference (Fig. 7). The figures show a trend of more preference for MP3000 in noisy conditions but the difference was not statistically significant (chi-square = 1.5, df = 1, *P* = 0.2). Also, there was no significant effect of language on strategy preference.

## Energy consumption

**Figure 8:**
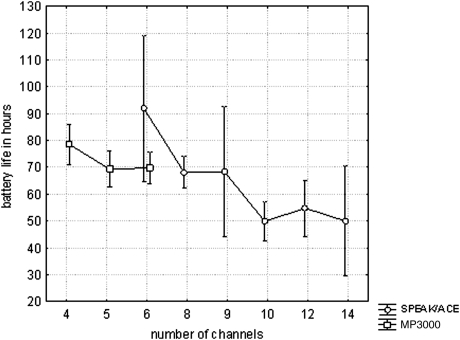
Battery life as a function of the number of channels used in the SPEAK/ACE and MP3000 strategies. The bars represent 95% confidence intervals. Large confidence intervals correspond to small number of cases.

**Figure 9:**
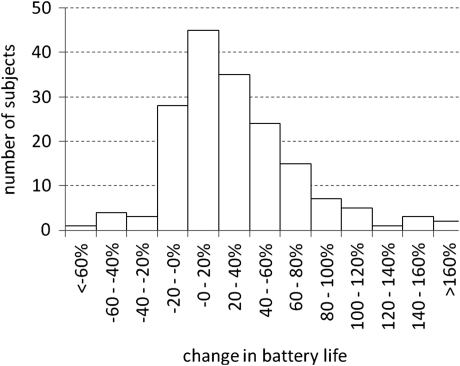
Distribution of change in battery life with change from SPEAK/ACE to MP3000 strategy across 172 subjects.

Battery life was assessed between sessions B1 and A2, and between A2 and B2, of the ABABA sequence. Comparing battery life between the MP3000 and SPEAK/ACE strategies showed a significant effect of the reduction in number of channels on battery life (*P* = 0.025, *F*(10,166) = 2.13). The effect is well illustrated while comparing battery life as a function of number of channels for SPEAK/ACE and MP3000 separately (Fig. 8). Linear regression of the data showed about 4 hours increase in battery life for one channel less. Battery life varied from about 20 to more than 100 hours. The increase in average battery life with smaller number of channels originates mainly with an increase in the longer periods. Both, for SPEAK/ACE and MP3000 shortest battery life of about 20 hours was found. The pair of slopes (50/37, 40/30, or 20/15 dB/Bark) did not have a significant effect on battery life (*P* = 0.14, *F*(2,200) = 2.0). The distribution of the increase in battery life across all subjects is presented in Fig. 9. With three Zinc-Air batteries the average increase in battery life was 24%.

## Discussion

The present study shows that there is no difference between the speech scores found for individually optimized SPEAK/ACE and MP3000 strategies. This result is found for speech scores collected both in a quiet and in a noisy background and moreover for speech scores collected in noise at S/N = +10 and 0 dB. Also, there are no differences between the two strategies for eight languages with their respective speech tests. In addition, strategy preference was about equally distributed between the SPEAK/ACE and MP3000 strategies (Figs 6–8).

Equal scores were found for the two strategies while the number of channels was 6–14 in SPEAK/ACE and only 4–6 in MP3000. Since the pulse rate per channel was not changed when switching from SPEAK/ACE to MP3000, it implied that equal scores were found for markedly lower total pulse rates in MP3000 than in SPEAK/ACE. This is reflected in battery life, which for MP3000 was 24% longer with three Zinc-Air batteries than for SPEAK/ACE.

The present study did not include a SPEAK/ACE condition with 4–6 channels. Thus, it does not prove that the MP3000 coding principle accounts for maintaining the scores while reducing the number of channels. However, a previous study has shown that at least eight channels are required to achieve maximum speech scores with the ACE strategy (Plant *et al*., 2002). Also, other SPEAK or *N*-of-*M* studies in which both the number of channels and channel rate were varied suggest that maximum speech scores are reached with at least 8–10 channels (Fishman *et al*., 1997; Ziese *et al*., 2000; Friesen *et al.*, 2001). Moreover, Buechner *et al*. (2008) showed for the Oldenburg sentence test that ACE with eight channels yielded significantly higher (worse) thresholds than an MP3000 type of coding scheme with eight channels and even with four channels. Thus, the high scores found for MP3000 may be attributed to its coding principle that results in more dispersion, less clustering of the channels stimulated. These results might suggest that MP3000 could yield even better results than ACE for number of channels higher than eight. However, the advantage of more dispersion in the channels sampled decreases as the number of channels increases. Obviously, there is no difference between the two coding strategies if all channels (*M*-of-*M*) are used. Kals *et al*. (2010) found that the number of channels could be reduced from 11 to 4 without losing speech performance in noise if forcing the selection of the smaller number of selected frequency components into a wider spectral distribution (less cluttering). This technique is computationally less intensive. However, it forces the selection of channels into a wider spectral distribution according to a fixed preset scheme, not according to a physiologically based masking function operating dynamically.

The MP3000 strategy was individually optimized choosing among 4, 5, or 6 channels and three combinations of slopes: 50/37, 40/30, and 20/15 dB/Bark. Since it was considered to be too demanding for the subject to start with nine conditions to choose from, it was decided to determine the individual's optimum in two steps: starting by determining the optimum number of channels with one fixed pair of slopes assigned at random and subsequently determining the optimum pair of slopes for the optimum number of channels found in the first step. Tables 1 and 2 showed that there was no effect of the preset pair of slopes on either the number of channels with the highest score or the preferred number of channels. Although individual dependence of the best number of channels on the preset pair of slopes cannot be excluded, this result suggests that the preset values had little effect on the resulting best number of channels. The next step showed a similar result: the optimum pair of slopes did not depend on the best number of channels found in the previous step (Table 3). Comparing the best pair of slopes with the preset values shows quite a shift toward the steeper slopes (Table 4). Together these results strongly suggest that the two-step approach toward finding the best combination of number of channels and pair of slopes, starting with preset slopes, did not introduce undesired interactions in the procedure.

For all preset pairs of slopes, the preferred number of channels was evenly distributed for the speech scores collected in quiet but the larger number of channels showed better performance for sentences presented in noise and there was a clear preference for the higher number of channels. The rate per channel was independent of the number of channels. Thus, more channels implied a higher total rate. Both, total rate or number of channels could have yielded better speech information transfer. The present result suggests a default value of six channels.

The result for the best pair of slopes is intriguing, although in line with the results of pilot experiments (e.g. Nogueira *et al*., 2005). The speech scores are highest for the steepest slopes and these slopes are also preferred. This implies that one prefers the condition with the smallest amount of spectral spread. This might be a habituation result. The subjects were accustomed to the SPEAK/ACE strategy for a median period of 1.4 years (Fig. 2). However, this finding may also be related to the dispersion of the selected spectral components. With shallower slopes there is more dispersion resulting in more spectral peaks represented by two or only one frequency component (see Nogueira *et al*., 2005, Table 2). This representation may jump across channels from one spectral sample to the next, which could produce a noisier percept. Still, it is not quite understood why such steep slopes are preferred whereas the estimates of spread of excitation are much broader (Abbas *et al*., 2004; Cohen *et al*., 2004, 2005). Most estimates of spread of excitation are based on the old straight array electrode. One might assume smaller spread of excitation for the new Contour™ electrode. Cohen *et al*. (2005) did not find significant differences in spread of excitation between these two types of electrodes, whereas van Weert *et al*. (2005) and Cohen (2009) found slightly less spread of excitation for the Contour electrode but the results do not suggest that the adequate masking function for MP3000 should have slopes steeper than the typical psychoacoustical slopes of 24 dB/Bark (Zwicker and Feldtkeller, 1967; Zwicker and Fastl, 1990). Yet, the present result suggests that one could use the 50/37 dB/Bark combination as the default value. Steeper slopes were not included in the present study. Thus, it cannot be excluded that steeper slopes might have yielded even better results. However, in view of the above discussion this is not expected.

In conclusion, the new MP3000 coding strategy, taking into account spread of masking, allows for a reduction in number of channels from typically 8–12 in conventional coding (SPEAK/ACE) to 4–6 in MP3000 without losing performance. The reduction in number of channels implies 24% increase in battery life. If one is interested in increasing performance rather than in increasing battery life one might try to use more than 4–6 channels in MP3000. However, the increase in performance will be limited because the difference between the two coding strategies will decrease as the number of channels increases. The best speech scores were found for the steepest masking slopes applied in the MP3000 coding strategy. This result was not quite expected. It was discussed above but future research should reveal the optimum steepness of the slopes and it should clarify the relation between the optimum steepness and known estimates of spread of masking and spread of excitation.

## Glossary

ACE: advanced combinational encoder, similar to SPEAK but with higher pulse rates

CIS: continuous interleaved sampling, a strategy in which a fixed number of frequency channels is sampled continuously in a fixed order

MP3000: a coding strategy in which only *N* channels of *M* available channels are chosen dynamically according to the instantaneous peak levels in all channels relative to a physiologically-based masking function

*N*-of-*M*: a coding strategy in which only *N* channels of *M* available channels are chosen dynamically

SPEAK: spectral peak strategy, a strategy in which only *N* channels of *M* available channels are chosen dynamically according to the instantaneous peak levels in all channels
